# C.35delG/ *GJB2 *and del(*GJB6*-D13S1830) mutations in Croatians with prelingual non-syndromic hearing impairment

**DOI:** 10.1186/1472-6815-5-11

**Published:** 2005-12-08

**Authors:** Igor Medica, Gorazd Rudolf, Manuela Balaban, Borut Peterlin

**Affiliations:** 1 Division of Medical Genetics, Department of Obstetrics and Gynaecology, University Medical Centre Ljubljana, Slovenia, Slajmerjeva 3 1000 Ljubljana, Slovenia; 2 Outpatient Paediatric Clinic Pula, Croatia, Istarska 13 52100 Pula, Croatia

## Abstract

**Background:**

C.35delG/GJB2 mutation is the most frequent genetic cause of deafness in Caucasians. Another frequent mutation in some Caucasian populations is del(*GJB6*-D13S1830). Both *GJB2 *and *GJB6 *genes belong to the same DFNB1 locus and when the two mutations are found in combination in a hearing-impaired person, a digenic pattern of inheritance is suggested.

**Methods:**

We examined 63 Croatian subjects (25 familial and 38 sporadic cases) with prelingual non-syndromic hearing impairment by polymerase chain reaction for the presence of the c.35delG/*GJB2 *and the del(*GJB6*-D13S1830) mutations.

**Results:**

Of the 63 unrelated hearing-impaired subjects, the mutation c.35delG/*GJB2 *was found in 21 subjects (33.3%). In 5 of them the mutation was found in the heterozygous state, all of them being compound heterozygotes, as sequencing revealed a second mutation within the coding region of the gene in 3 subjects, and a splice site mutation in 2 subjects. The del(*GJB6*-D13S1830) mutation was not found in the investigated hearing-impaired Croatian subjects.

**Conclusion:**

Our results contribute to the knowledge of geographic distribution and population genetics of the *GJB2 *and *GJB6 *mutations in the Europeans.

## Background

The identification of genes causing non-syndromic hearing impairment has partially resolved the puzzle of clinical and genetic heterogeneity of deafness [1]. Among these genes the gene with the most significant impact on the population genetics and genetic counselling is the *GJB2 *gene with the mutation c.35delG that accounts for the majority of mutations in deaf Caucasians [1-3]. Studies published so far have reported the differences in frequency of the mutation in different populations, and its variability in clinical impact on hearing impairment [4,5]. However, although the mutations in the *GJB2 *gene are responsible for up to 69% of autosomal recessive non-syndromic hearing impairment [2], a problem emerges when patients are identified with only one *GJB2 *mutant allele. Recently, in the same DFNB1 locus, a 309-kb deletion implicating the *GJB6 *gene, del(*GJB6*-D13S1830), has been identified, and was found to be very common in non-syndromic hearing-impaired patients from Spain, France, Israel, the United Kingdom and Brazil, suggesting also a possible *GJB2 */ *GJB6 *digenic pattern of inheritance of deafness [6,7]. There are no reports on frequencies of the c.35delG/*GJB2 *and del(*GJB6*-D13S1830) mutation in either the hearing impaired in Croatia, or in the Slavic populations of Central-Eastern Europe.

The aim of our study was to establish the frequencies of c.35delG/*GJB2 *and del(*GJB6*-D13S1830) mutations in Croatians with prelingual non-syndromic hearing impairment.

## Methods

Patients with hearing impairment were ascertained through two regional otorhinolaryngology centres (General Hospital Pula and University Medical Centre Rijeka). The inclusion criteria were: prelingual, non-syndromic hearing loss of unknown aetiology. Both the patients with familial history of deafness and sporadic cases were included. The study group consisted of 66 patients: 29 males and 37 females, with ages ranging from 3 months to 73 years; 28 cases had familial history of deafness, 38 were sporadic. Of the 66 subjects, 63 were unrelated.

Fifty adult patients were audiologically evaluated by pure tone audiometry with a diagnostic audiometer in a soundproof room according to the International Organization for Standardization (ISO). The conductive component of hearing loss was excluded by tympanometry. The air conduction binaural mean pure tone threshold (dB hearing loss) was recorded for 500, 1000, 2000, and 4000 Hz. Hearing impairment was classified as mild when the average threshold was between 21 and 40 db, moderate if 41–70 db, severe if 71–95 db, and profound if more than 95 db. In 16 children, aged from 3 months to 16 years, the testing was performed by auditory brainstem response [8,9].

After DNA isolation from the peripheral blood by standard procedures, all patients were molecularly evaluated for the presence of the c.35delG mutation by allele-specific polymerase chain reaction method (PCR) [10]. Sequencing was performed in patients heterozygous for the mutation at GENDIA – Genetic Diagnostic Network, Antwerp, Belgium. All patients were also evaluated for the presence of del(*GJB6*-D13S1830) mutation by the PCR method described by del Castillo et al [7].

The study was approved by the institutional medical ethics committee, and was performed after the informed consent of the patients or their parents was obtained.

## Results

### Mutation analysis

Of the 63 unrelated investigated patients, the c.35delG/*GJB2 *mutation was found in 21 subjects (33.3%): 12 males and 9 females. In 16 out of 63 subjects the mutation was found in the homozygous state, in 5 in the heterozygous state. In the latter cases, sequencing of the coding exon, in search for the second mutation was performed. In three patients the sequencing revealed, besides the c.35delG mutation, three different second mutations already known as pathogenic: c.235delC, p.E47X and p.R143W respectively. In remaining two patients the sequencing revealed a known splice site mutation which is also already disease-associated: c.IVS1+1G>A.

Twelve homozygous and 1 heterozygous patients out of the 21 had a positive familial history of deafness, 4 homozygous and 4 heterozygotes had no familial history of deafness. In the group of 25 subjects with positive familial history, 12 were homozygous for the c35delG mutation (48%), 1 heterozygous (4%), while in the group of 38 sporadic cases 4 were homozygous (12.5%) and 4 heterozygous (12.5%).

None of the 63 patients was found to carry the mutation del(*GJB6*-D13S1830) in either the homozygous or heterozygous state.

### Phenotypic analysis

When considering all 66 investigated patients, the c.35delG/*GJB2 *mutation was found in 24: 13 males and 11 females. In 19 out of 66 subjects the mutation was found in the homozygous state, in 5 in the heterozygous state. Of the 19 patients with hearing impairment due to the c.35delG/*GJB2 *mutation, 16 had profound deafness, one had severe, and two had moderate hearing loss. All patients had sensorineural bilateral hearing loss. All but 2 patients had symmetric hearing impairment. The 3 patients – compound heterozygotes: c. [35delG] + [235delC], c. [35delG] + p. [E47X], c. [35delG] + p. [R143W] had also profound deafness while 2 patients compound heterozygotes c. [35delG] + [IVS1+1G>A] had severe and moderate deafness respectively.

## Discussion

The c.35delG mutation in the *GJB2 *gene has been identified as the most important among genetic causes of hearing impairment as it accounts for the majority of mutations in deaf Caucasians, whether in the homozygous state or as one of the mutations in compound heterozygosity (within *GJB2 *or in *GJB6 *gene) [4,7]. The studies have been published reporting the frequency of the mutation in different populations showing a south-to-north European gradient, where a high prevalence of the mutation in the populations of Southern Europe has been explained by the founder effect [4,5,11,12]. The share of the c.35delG involvement in hearing impairment varies from 28 to 63% [4]. There are only a few reports on its frequency in the populations of Slavic origin [13,14], but no mutation frequency data have been published on the Croatian or other Slavic Central-Eastern European populations. The del(*GJB6*-D13S1830) mutation has been identified as the second most frequent mutation causing non-syndromic, prelingual, autosomal recessive hearing impairment in the European populations of Spain, France, the United Kingdom, but also in Brazilians and Jews [7]. Like the c.35delG mutation, this deletion is also population specific as it was established that it is very rare in Czech population, while it is absent in the Austrian population [15,16], showing a west-to-east European gradient and indicating again a founder effect as it is the case of c.35delG/*GJB2 *mutation. There are no reports on the share of this mutation in hearing impaired Slavic Central-Eastern European populations.

Our results suggest the importance of the mutation c.35delG in the Croatian population, the data being comparable to those from the neighbouring areas [4,17,18]. Besides, results such as ours suggest the introduction of molecular genetics diagnosis of the mutation into clinical practice with significant impact on genetic counselling. However, although the involvement of the mutation in the aetiology of deafness has been found in 21 out of 63 hearing-impaired Croatian subjects, a possibility should be mentioned that some GJB2-related cases of deafness due to two less prevalent GJB2 mutations should escape detection in our investigation.

In our study, 16 of the 19 subjects with the c.35delG mutation involvement had profound deafness, one had severe and two had moderate hearing impairment. The results are in agreement with the majority of studies on the severity of hearing impairment related to the mutation, where mainly the patients with severe to profound hearing loss are reported, due to an important truncation of the protein after deletion [19-21]. Patients with mild and moderate hearing loss have been rarely reported [22,23].

Our data on the c.35delG/*GJB2 *frequency in Croatia contribute to the geographic distribution of the mutation in Europe, confirming the south-to-north gradient.

On the other hand, our results on the involvement of del(*GJB6*-D13S1830) mutation in the aetiology of hearing impairment show that the mutation is of no epidemiological and clinical importance in Croatians, the results being in agreement with the results of the frequency of the mutation in populations from neighbouring areas, i.e. from Central European populations [14-16].

## Competing interests

The author(s) declare that they have no competing interests.

## Authors' contributions

IM recruited patients, performed data interpretation, and wrote the manuscript. MB and GR carried out the molecular genetic studies and participated in data interpretation. BP designed, coordinated, and supervised the study and reviewed the completed manuscript. All authors read and approved the final manuscript.

## Pre-publication history

The pre-publication history for this paper can be accessed here:


